# A Systematic Review on Effects of Nitrogen Fertilizer Levels on Cabbage (*Brassica oleracea var. capitata* L.) Production in Ethiopia

**DOI:** 10.1155/2024/6086730

**Published:** 2024-04-30

**Authors:** Yohannes Gelaye

**Affiliations:** Department of Horticulture, College of Agriculture and Natural Resources, Debre Markos University, P.O. Box 269, Debre Markos, Ethiopia

## Abstract

Cabbage (*Brassica oleracea* var. *capitata* L.) holds significant agricultural and nutritional importance in Ethiopia; yet, its production faces challenges, including suboptimal nitrogen fertilizer management. The aim of this review was to review the possible effect of nitrogen fertilizer levels on the production of cabbage in Ethiopia. Nitrogen fertilization significantly influences cabbage yield and quality. Moderate to high levels of nitrogen application enhance plant growth, leaf area, head weight, and yield. However, excessive nitrogen levels can lead to adverse effects such as delayed maturity, increased susceptibility to pests and diseases, and reduced postharvest quality. In Ethiopia, small-scale farmers use different nitrogen levels for cabbage cultivation. In Ethiopia, NPSB or NPSBZN fertilizers are widely employed for the growing of various crops such as cabbage. 242 kg of NPS and 79 kg of urea are the blanket recommendation for the current production of cabbage in Ethiopia. The existing rate is not conducive for farmers. Therefore, small-scale farmers ought to utilize an optimal and cost-effective nitrogen rate to boost the cabbage yield. Furthermore, the effectiveness of nitrogen fertilization is influenced by various factors including the soil type, climate, cabbage variety, and agronomic practices. Integrated nutrient management approaches, combining nitrogen fertilizers with organic amendments or other nutrients, have shown promise in optimizing cabbage production while minimizing environmental impacts. The government ought to heed suggestions concerning soil characteristics such as the soil type, fertility, and additional factors such as the soil pH level and soil moisture contents.

## 1. Introduction

Cabbage, a member of the *Cruciferae* (*Brassicaceae*) family, is a biennial plant often cultivated annually, characterized by a short stem and dense cluster of leaves forming a compact head [1]. It has been reported that they are closely related to broccoli, cauliflower, and Brussels sprouts and originate from Western Europe and the Northern shores of the Mediterranean [2]. Cabbage is grown in more than 90 countries worldwide [3]. China and India are typically the top producers, followed by South Korea, Germany, Japan, and South Africa [4]. South Africa, Kenya, Egypt, Ethiopia, and Niger are the top five cabbage producers on the African continent. It ranks fifth among vegetable crops worldwide [5]. Cabbage, a popular cool season crop among gardeners and commercial producers, stands as one of the most significant vegetable crops cultivated worldwide [6]. It has been domesticated since ancient times and is renowned for its nutritional value and abundant mineral and vitamin content, including vitamins A, B1, B2, and C, making it a staple in human consumption [7]. Cabbage is also known for its cooling effect as an appetizer that can help prevent constipation [8] and protect against cancer [9]. Although cabbage prefers cool and moist climates, it can grow easily under a wide range of environmental conditions in both temperate and tropical regions [10]. Cabbage, alongside chili, holds the distinction of being Ethiopia's second most vital vegetable crop, both in terms of widespread cultivation and production [5]. However, owing to the scarcity and high cost of chemical fertilizers, most small-scale farmers in the country employ fertilizer levels lower than the recommended rate [11]. While the ideal yield is determined by applying 79 kg/ha, there has not been enough research into how varying rates of N-fertilizer application affect the yield and yield components of cabbage [12]. Hence, the yield obtained was reported to be low (6–20 tons/ha) [13]. Similarly, declining produce quality and soil fertility in Ethiopia pose significant challenges for market acceptance and production. Nitrogen's role in cabbage yield varies with the soil type, soil fertility, and environmental factors. Research indicates that proper management of nitrogen application in terms of rate and timing can boost the cabbage yield [14]. To secure high cabbage yield, it is also important to use the optimum rate of nitrogen per unit area [15]. The ideal nitrogen rate crucially affects solar radiation absorption in cabbage crops. Despite global research, Ethiopia lacks specific studies on this aspect, which are impacted by local factors such as soil and climate. Understanding how varieties and conditions dictate optimal nitrogen levels is vital for sustainable cabbage farming, enabling the balancing of increased production with environmental concerns through tailored management practices [16]. Continuous high nitrogen use can harm soil health, necessitating long-term studies, while climate change necessitates resilient fertilizer strategies, adding complexity [17]. To benefit Ethiopian farmers, research must bridge the gap between findings and practical application through knowledge-sharing initiatives and extension services. Thus, addressing these gaps can enhance our understanding of nitrogen fertilizer's impact on cabbage production in Ethiopia, bolstering sustainable agricultural development in the region. Therefore, the aim of this review was to review the effect of nitrogen fertilizer levels on the growth and yield components of cabbage.

## 2. Review Methodology

**Figure 1:**
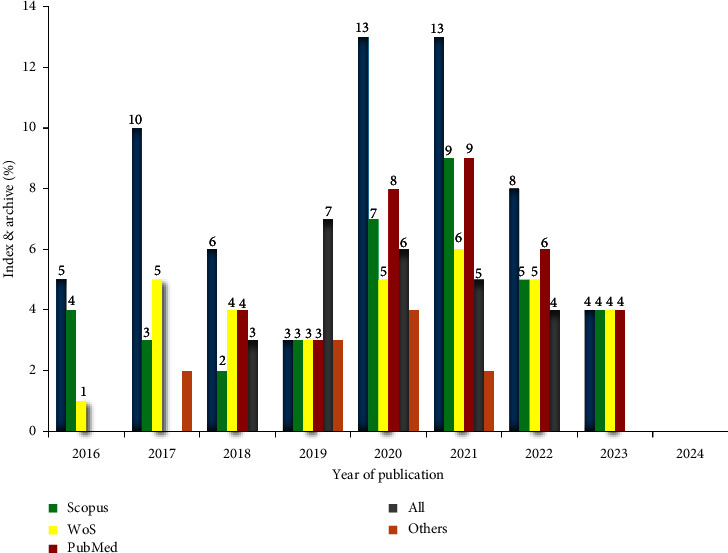
Graphical representations depicting tactics for gathering articles related to cabbage cultivation.

In this review of the literature, I employed diverse approaches. I referred to respected journals sourced from Scopus, Web of Science, and PubMed databases. The selection criteria predominantly targeted articles released post-2019, while omitting pertinent data and books (Figure 1). Therefore, the review materials were chosen spanning the years, guided by indices and respective protocols. In total, 32 articles (30.4%), 37 articles (35%), 34 articles (32%), 25 articles (23%), and 37 articles (35%) were sourced from WoS, Scopus, PubMed, All, and other databases, respectively. The year-wise analysis indicates that the majority of articles were published between 2019 and 2023 (Figure 1). The majority of the reviewed papers concluded that varying levels of nitrogen fertilizer significantly impact the growth, yield, and quality components of cabbage crops.

## 3. Origin and Distribution of Cabbage

Cabbage (*Brassica oleracea* var. *capitata*) is believed to have originated from the Mediterranean region, specifically from the wild cabbage plant native to coastal areas of Europe [3]. It has a long history of cultivation, dating back thousands of years [18]. In Ethiopia, cabbage is thought to have been introduced during the colonial period, primarily by European settlers and missionaries [19]. Cabbage cultivation in Ethiopia has grown, mainly in highland regions due to favorable climate [20]. These regions encompass the central highlands, including Addis Ababa, and the northern highlands, home to cities such as Bahir Dar and Gondar. Cabbage cultivation in Ethiopia has increased significantly in recent years due to its adaptability to various agroecological zones, its nutritional value, and its economic importance [21]. It is cultivated both by small-scale farmers for local consumption and by larger commercial farms for export markets [22]. Overall, cabbage significantly contributes to Ethiopian agriculture and diet, enhancing food security and farmer income nationwide.

## 4. Description of Cabbage

Cabbage has a chromosome number of 2*n* = 2*x* = 18 and has been grown for more 3000 years in the Mediterranean region [23]. It is described as originating in Western Europe, with temperate climates, and a kale-like ancestor was grown in gardens as far back as the time of the Roman Empire [24]. Cabbage is described as a biennial crop although it is grown as an annual crop, and there is a great difference between the cultivated types of cabbage [25]. It is also known to have an adventitious root system and an unbranched stem that remains less than 30 cm long [26]. As cabbage grows, leaves multiply to form a ball-shaped “head” at the plant's centre, primarily composed of a large vegetative terminal bud resulting from a leaf overlying its shortened stem [27]. An example from a 2010 Ethiopian guide on cool season vegetable crops, seed production, and development practices describes head cabbage (common cabbage) as having a dense head of leaves, a short stem, and additional edible leaves [28]. The diverse shapes and sizes of cabbage heads across varieties encompass oblong, oval, or nearly circular forms, with cultivars displaying distinctions in the leaf size, shape, color, and head texture [29]. Clear descriptions of cabbage growth stages, though lacking standard terminology, are vital for pest management, as plant susceptibility varies, as outlined in subsequent literature (Table 1).

## 5. Cabbage Production in Ethiopia

The annual production of cabbage in the world is estimated to be about 70,644,191 tons with an average productivity of about 29.23 tons/ha [30]. The report from the Central Statistical Agency (CSA) in Ethiopia details 5150.14 hectares of land, yielding 35,123.65 tons of produce with a productivity rate of 6.8 tons per hectare, falling below the global average [31]. Cabbage is frequently produced during the rainy season although some commercial farmers produce it during the dry season using irrigation [32]. In Ethiopia, cabbage is cultivated both in mixed cropping systems and monoculture, with irrigation noted to significantly increase cabbage yield compared to rain-fed conditions. Cabbage cultivation is scattered throughout the highlands of Ethiopia. Cabbage production in Ethiopia centers on Copenhagen and early drum head varieties in the central highlands, favored by urban landholders and characterized by shallow depth, contrasting with rural farmers.

## 6. Importance of Cabbage

**Figure 2:**
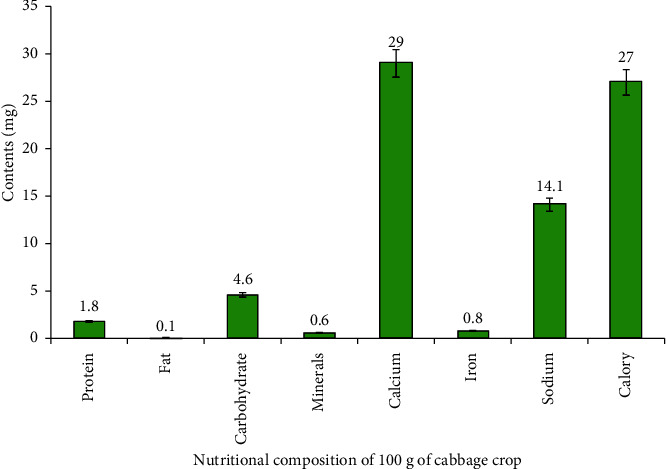
Nutritional composition of cabbage [6].

Cabbage is an economically and nutritionally important vegetable crop worldwide and is one of the most extensively consumed vegetable crops in Ethiopia, particularly in urban areas [33]. It has been reported as one of the top 20 vegetables used as a global food source [34]. Cabbage, prized for its leaves, is a rich source of minerals such as calcium, iron, sodium, potassium, and phosphorus, as well as essential vitamins, proteins, and carbohydrates [6]. Head cabbages have significant quantities of nutrients and for instance, a 100 g edible portion of cabbage is reported to contain 27 mg of calories, 1.8 mg protein, 0.1 mg fat, 4.6 mg carbohydrate, 0.6 mg mineral, 29 mg calcium, 0.8 mg iron, and 14.1 mg sodium (Figure 2). Cabbage, rich in beta-carotene and ascorbic acid, also contributes to a healthy diet with glutamine, amino acids, and dietary glucosinolates, supporting human health [35]. Similarly, it has also been employed in the preparation of soup mixtures with other vegetables, such as carrots, celery, corn, and onions [36]. Currently, consumers in Ethiopia are aware of about cabbage crops, which are becoming more prevalent because of their nutritional value and anticancer properties.

## 7. Ecological Requirements of Cabbage

**Figure 3:**
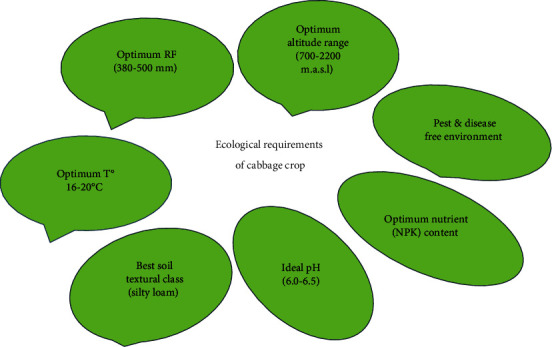
Ecological necessities of cabbage crop.

Cabbage is grown within an altitude range of 700–2,200 m.a.s.l [37]. In low-altitude regions, crop cultivation, particularly of water-intensive crops like cabbage, should be timed for the cooler months of the year to align with Ethiopia's specified rainfall requirement of 380–500 mm, evenly distributed throughout the growing period [10]. Cabbage's water needs vary with soil, growth stage, and environmental factors, thriving in cooler temperatures with an optimal production range of 16–20°C (Figure 3) [38]. Temperatures exceeding 250°C have been observed to impede head formation in cabbage, which thrives in well-drained sandy or silty loam with a high organic matter content [39]. Correspondingly, the optimal soil pH range for cabbage cultivation is 6.0–6.5 [40]. Cabbage, like most leafy vegetables, is considered a heavy feeder and does well in soils with high nitrogen content [41]. Nitrogen, phosphorus, and sodium are vital for cabbage growth, with nitrogen specifically noted for fostering rapid growth, high yield, and quality [42]. In addition, the level of nitrogen is reported to lead to a higher dry matter content in the shoots than in the roots [43]. Experts conducting research identified a strong correlation between nitrogen fertilizer application and cabbage quality, recommending 242 kg of NPS and 79 kg of urea for cabbage cultivation in Ethiopia [44]. Cabbage is harmful to light frost and is attacked by many pests and diseases (Figure 3) [45]. Typically, cabbage is transplanted and shows a good response to nitrogen [46].

## 8. The Effect of Nitrogen on the Cabbage Yield

Cabbage growers in Brazil use blanket fertilizer recommendations, including nitrogen, at different rates and times of application [47]. Cabbage is a highly demanding vegetable crop for major plant nutrients, including nitrogen. The doubling of agricultural food production worldwide over the past four decades has been linked to a sevenfold increase in the use of nitrogen fertilizers [48]. The ongoing discovery of nitrogen fertilizers in agriculture will significantly impact the industry, exemplified by cabbage absorbing nitrogen from organic matter such as nitrate or ammonium [49]. Nitrogen is reported to play a role in protein formation as a component of chlorophyll; thus, an adequate supply of nitrogen increases the efficiency of chlorophyll [50]. In Bangladesh, from 2013 to 2014, a study assessed the impact of varying nitrogen doses on cabbage growth and yield, suggesting that utilizing 200 kg N/ha could be beneficial for cabbage cultivation [51]. If a plant is supplied with an optimum amount of nitrogen, there is a tendency to increase leaf cell number and cell size, with an overall increase in leaf production. Moreover, the cabbage cultivation manual indicates that cabbage requires plenty of NPK nutrients for head formation [52]. In Pakistan, application of NPK farmyard manure (FYM) at a ratio of 160 : 90 : 60 along with 15–20 tons/ha of farmyard manure is reported to have a desirable effect on the growth and marketable yield of cabbage [53]. A study conducted to evaluate the impact of increasing plant populations combined with different doses of nitrogen on the yield of cabbage, its structure, and plant composition indicated that the enhancement of N dose to 300 kg N/ha was beneficial for the total yield of heads as well as those weighted greater than 1.0 kg [54]. The sole alteration in plant composition due to heavy nitrogen fertilization was the rise in nitrate accumulation and reduction in calcium content in cabbage heads, emphasizing that optimal nitrogen and nutrient levels ensure a well-balanced mineral nutrition profile [55]. Imbalanced nitrogen application harms cabbage quality, causing loose-head formation and decay; however, employing techniques such as high-density planting and careful fertilization can help control the size. A field experiment conducted to evaluate the potential of using bioslurry as organic manure for cabbage production in Bangladesh stated that it is possible to reduce 30% of the recommended nitrogen and K fertilizers and 100% of the phosphorus and sulfur fertilizers through the use of poultry or cow dung bioslurry in cabbage production [56, 57]. Research on Chinese cabbage in organic farming showed significant impacts on growth, yield, and quality by combining bioslurry, derived from anaerobic decomposition of organic materials, with inorganic fertilizers, thereby sustaining crop production and enhancing soil health [58, 59]. Manure application can reduce invalid crop water consumption, use soil water reasonably, and improve water use efficiency at different growth stages of a crop.

An experiment on head cabbage yield and quality found that decreased N and K residuals postharvest suggested complete fertilizer absorption, which is crucial ecologically [60]. Organic manure and biofertilizers improve soil health and crop quality, as evidenced by a study on red cabbage cultivation assessing nutrient efficiency, productivity, and soil sustainability [15]. In addition, a field experiment was conducted to evaluate the growth, productivity, and economics of cabbage in the grid zone as influenced by different levels of zinc and sulfur reported that growth parameters such as plant height, plant spread, number of leaves per plant at all crop growth stages, yield attributing characters, length and diameter of head and weight per head, cabbage yield per hectare net return, and benefit cost ratio increased with increasing levels of sulfur from 20 to 80 kg/ha and zinc from 2 to 8 kg/ha and the crop responded only up to 60 kg *S/*ha and 6 kg Zn/ha [61, 62]. During a 2017 experiment in Ghana's Kintampo North Municipality, evaluating cabbage response to various soil amendments from August to November, poultry manure matched NPK (15-15-15) and outperformed other treatments across all measured parameters, while cow dung and goat manure also exhibited promising results [63, 64]. Likewise, a field experiment conducted to determine the optimum combination of FYM and biofertilizers for improving the growth, yield, and quality attributes of Chinese cabbage under high-altitude rain-fed climatic conditions reported that among the treatments studied, the highest plant height (33.33 cm), number of wrapper leaves/plant (18.00), plant spread/plant (111.66 cm), head weight/plant (553.33 g), yield/plot (15.49 kg), yield/ha 34.42 tons, moisture content 98.00%, and vitamin C 28 mg/100 g were observed with the application of FYM at 25 tons/ha along with an azotobactor and phosphate-solubilizing bacteria [65].

An experiment was conducted to evaluate the performance of three cabbage varieties (*Brassica oleraceae* var. *capitata* L.) varieties (Benelli', “Cairo” and “Paradox”) (Bahamas-2012) indicated that the variety “Paradox” produced the longest head length and the largest head width [66]. The variety produced the highest head weight and best yield potential of 37.7 tons/ha, while combining vermicompost with integrated plant nutrients and recommended chemical fertilizers in a field experiment resulted in superior cabbage growth and yield compared to using chemical fertilizers alone [67].

During field trials conducted at the Umsunduze Training Centre in KwaZulu Natal in 2005 and 2006, exploring the effects of three organic fertilizers (chicken, Kraal manure, and compost) on cabbage (Conquistador cultivar) and carrots (Kuroda cultivar) growth, yield, and quality, it was found that cabbage head diameter was consistent between those treated with chicken manure and inorganic fertilizer [68]. A study on cabbage yield and head weight revealed that, on average, heads weighed 3.8 pounds, with the lowest marketable yield observed in plants spaced at 9 inches and receiving low fertilizer rates [69].

A research designed to carry out a two-season trial on two cabbage (*Brassica oleracea* var. *capitata* L.) cultivars in spring and autumn in the Beijing–Tianjin–Hebei region (2017) stated that the application of drip irrigation under mulch should be approximately 114.7–125.0 mm and the N fertilization should be about 200 kg/ha [70]. In addition, a study conducted to determine the effect of fertilizer type on the growth and yield of two cabbage varieties (Copenhagen market and F1 minor) in Nigeria (2009 and 2010) reported that compared with NPK (15 : 15 : 15), NPK enhanced the optimum yield of cabbage varieties [71]. Moreover, the Copenhagen market variety outperformed F1 minor in head yield regardless of fertilizer application, making it the preferred choice in Ogbomoso, Southwest Nigeria, with field experiments demonstrating that fertilizing cabbage plants with 80 kg N/fed alongside 100 ppm fulvic acid results in high-quality heads suitable for local consumption in both growing seasons [72].

Combining bioslurry and NP inorganic fertilizers in southern Ethiopia significantly enhanced cabbage growth, with the study recommending the application of 50 m^3^/ha of bioslurry alongside 75% NP for optimal production under similar conditions [73]. Furthermore, a study conducted to assess the response of head cabbage to different rates of inorganic nitrogen fertilizer and farmyard manure in Bore, Southern Oromia, Ethiopia, reported that head cabbage responded well to the combined application of N and FYM and that application of 235 kg N/ha +6 tons FYM/ha can provide the optimum cabbage head yield in the study area [13]. Nitrogen levels profoundly affect the cabbage yield by supporting crucial growth processes such as leaf and stem development, chlorophyll production, and overall plant vigor [74]. When cabbage plants receive insufficient nitrogen, they may exhibit stunted growth, reduced leaf size, and pale green foliage [75]. Excessive nitrogen can result in overly lush vegetative growth, potentially reducing yields and head size, while simultaneously increasing susceptibility to pests and diseases [76]. Optimizing cabbage yields requires testing soil for nitrogen levels and adjusting fertilizer applications accordingly to maximize growth [77]. In addition, the timing and method of nitrogen application can also influence cabbage growth and yield [78]. Split applications throughout the growing season may help maintain more consistent levels of available nitrogen for the plants [79]. The impact of nitrogen levels on cabbage yield depends on factors such as the soil type, climate, cabbage variety, and farming methods. Understanding cabbage nitrogen uptake can optimize application timing and amount [80]. Research may investigate uptake kinetics, nitrogen efficacy for sustainable practices, environmental consequences like runoff and emissions, and nutrient interactions for yield optimization [81]. In general, nitrogen is crucial for cabbage growth and yield, but proper management is a key to optimize results and prevent harm to plant health and productivity.

## 9. Effect of Nitrogen Levels on the Growth of Cabbage

Soil fertility, especially nitrogen levels, significantly affects cabbage production in Ethiopia, with regional variations impacting it differently, while climate conditions such as temperature and rainfall also influence nitrogen availability and uptake by cabbage plants [82]. Ethiopia has diverse climate zones, ranging from highlands to lowlands, and has an impact on nitrogen availability [83]. The cabbage crop's growth depends on nitrogen fertilizer, with too little causing stunted growth and excess leading to lush foliage but poor root development and disease susceptibility [84]. The source of nitrogen fertilizer (e.g., urea, ammonium nitrate, and organic sources) can influence its availability to cabbage plants and the rate of nitrogen release in the soil [49]. Different cabbage varieties may have varying nitrogen requirements and responses to nitrogen levels in the soil [85]. Different cabbage varieties vary in nitrogen utilization efficiency, influenced by farming techniques such as irrigation, mulching, crop rotation, and intercropping, affecting nitrogen availability and uptake [86]. Nitrogen levels can influence the susceptibility of cabbage plants to pests and diseases [87]. High nitrogen levels can sometimes make plants more vulnerable to certain pests and diseases [88]. Environmental stresses such as drought or waterlogging can disrupt cabbage growth by affecting nitrogen uptake, with nitrogen application methods and cover crops playing vital roles in mitigating these effects [89]. Nitrogen interacts with other essential nutrients such as phosphorus, potassium, and micronutrients [90]. Imbalances in nutrient levels can affect cabbage growth and development. Therefore, comprehending and enhancing these elements could assist Ethiopian farmers in enhancing cabbage yields by efficiently controlling nitrogen levels in their fields.

## 10. Effect of Nitrogen Levels on Cabbage Quality

The significant and well-documented influence of nitrogen levels on cabbage quality underscores its crucial role as a nutrient essential for optimal growth and development in cabbage plants [91]. Adequate nitrogen levels result in larger, greener leaves, which contribute to the overall size and appearance of the cabbage head [92]. Cabbage plants supplied with ample nitrogen, a crucial constituent of chlorophyll vital for photosynthesis, generally yield higher, as it enhances the plant's energy production and overall yield potential [93]. The availability of nitrogen directly impacts the size and firmness of cabbage heads, with insufficient levels leading to smaller heads with loose leaves and excessive levels causing overly large but less compact heads [32]. Adequate nitrogen boosts cabbage's nutritional quality by spurring the synthesis of proteins, vitamins, and essential nutrients, enriching its overall value [94]. Optimal nitrogen levels bolster cabbage plants against pests and diseases by fostering robust growth and enhancing their ability to fend off attacks [95]. Maintaining a balanced nitrogen application is crucial to prevent delayed maturity, increased susceptibility to diseases, and environmental pollution via groundwater leaching [96]. In addition, the timing and method of nitrogen application play a crucial role in optimizing cabbage quality and minimizing potential negative impacts [42].

## 11. Chemical Composition of Nitrogen Fertilizer

Nitrogen fertilizers can come in various forms, each with its own chemical composition. Urea is a solid nitrogen fertilizer with a high nitrogen content, typically around 46% [97]. It is made up of carbon, nitrogen, oxygen, and hydrogen atoms [98]. Urea, rich in nitrogen, is a cost-effective fertilizer commonly used for cabbage plants in Ethiopia [99]. Ammonium nitrate (NH_4_NO_3_), a crystalline salt compound, comprises nitrogen in both ammonium and nitrate forms, constituting approximately 34% of the nitrogen content [100]. Ammonium sulfate ((NH_4_)_2_SO_4_) fertilizer contains both nitrogen and sulfur, and it is composed of nitrogen, hydrogen, sulfur, and oxygen atoms [101]. Anhydrous ammonia (NH_3_) is also a gas, not a solid like the others, and is often applied directly to the soil [102]. In general, these are some of the main nitrogen fertilizers used in agriculture, each with its own chemical composition and properties.

## 12. Summary and Conclusions

Cabbage (*Brassica oleracea* var. *capitata* L.) plays a pivotal role in Ethiopian agriculture, but its productivity hinges on nuanced factors, notably nitrogen fertilizer levels. Cabbage cultivation in Ethiopia grapples with nitrogen fertilizer management challenges, balancing the need for enhanced growth and yield against the risks of adverse effects from both excessive and moderate to high levels of nitrogen application, impacting overall yield and quality. Also, integrated nutrient management approaches, combining nitrogen fertilizers with organic amendments or other nutrients, show promise in optimizing cabbage production while minimizing environmental impacts. However, there is a lack of specific studies on how nitrogen fertilizer levels affect cabbage production in Ethiopia, indicating a need for further research. Bridging the gap between research findings and practical application, particularly in optimizing cabbage production through integrated nutrient management approaches tailored to local conditions, is crucial for supporting sustainable agricultural development in Ethiopia.

## 13. Recommendations

Design controlled experiments to evaluate varying nitrogen fertilizer levels' effects on cabbage yield, quality, and environmental impact in Ethiopian growing conditions using randomized controlled trials with replicated plots. Utilize isotope tracing to track nitrogen uptake by cabbage plants and understand nitrogen use efficiency. Establish long-term field trials to monitor effects on soil fertility, productivity, and environmental outcomes over multiple growing seasons. Employ a multidisciplinary approach integrating agronomy, soil science, environmental science, economics, and social sciences to comprehensively address nitrogen fertilizer management complexities. Engage stakeholders, including farmers and researchers, by integrating indigenous knowledge and traditional practices to ensure relevance and participation in research. Conduct economic analyses and develop tools to demonstrate nitrogen fertilizer management strategies' viability, while promoting knowledge exchange for improved practices.

## Figures and Tables

**Table 1:** Growth stages of cabbage.

S/n	Growth stages	Descriptions	References
1	Stage 1: cotyledons (Seed leaves)	No true leaves present	[103–105]
2	Stage 2: seedling	Up to 5 true leaves	
3	Stage 3: 6–8 true leaves	Ready for transplanting	
4	Stage 4: 9–12 true leaves	Base of stem still visible from above	
5	Stage 5: precupping	Approximately 13–19 leaves	
6	Stage 6: cupping	Approximately 20–26 leaves	
7	Stage 7: early head formation	Head diameter will be approximately 10 cm	
8	Stage 8: head fill	Head diameter will be approximately 10–20 cm, and a firm round head is visible within the wrapper leaves	
9	Stage 9: mature	Head diameter will be approximately 15–30 cm, and no new visible leaf production will occur after the head has attained maximum hardness and size. The head is reported to be ready for harvest and may split if not harvested in time	

## Data Availability

Data sharing is not applicable to this article as no new data were analyzed in this study.
